# Metacommunity structuring in stream networks: roles of dispersal mode, distance type, and regional environmental context

**DOI:** 10.1002/ece3.834

**Published:** 2013-10-14

**Authors:** Mira Grönroos, Jani Heino, Tadeu Siqueira, Victor L Landeiro, Juho Kotanen, Luis M Bini

**Affiliations:** 1 Ecosystem Change Unit, Finnish Environment InstituteOulu, Finland; 2 Department of Biology, University of OuluOulu, Finland; 3 Departamento de Ecologia, Universidade Estadual Paulista – UNESPRio Claro, Brazil; 4 Departamento de Botânica e Ecologia, Universidade Federal do Mato GrossoCuiabá, Brazil; 5 Departamento de Ecologia, Universidade Federal de GoiásGoiás, Brazil; 6 Centre for Economic Development, Transport and the Environment for South SavoMikkeli, Finland

**Keywords:** Active dispersal, aquatic macroinvertebrates, community structure, environmental filtering, headwater streams, Moran's eigenvector maps, partial redundancy analysis, passive dispersal, variation partitioning

## Abstract

Within a metacommunity, both environmental and spatial processes regulate variation in local community structure. The strength of these processes may vary depending on species traits (e.g., dispersal mode) or the characteristics of the regions studied (e.g., spatial extent, environmental heterogeneity). We studied the metacommunity structuring of three groups of stream macroinvertebrates differing in their overland dispersal mode (passive dispersers with aquatic adults; passive dispersers with terrestrial adults; active dispersers with terrestrial adults). We predicted that environmental structuring should be more important for active dispersers, because of their better ability to track environmental variability, and that spatial structuring should be more important for species with aquatic adults, because of stronger dispersal limitation. We sampled a total of 70 stream riffle sites in three drainage basins. Environmental heterogeneity was unrelated to spatial extent among our study regions, allowing us to examine the effects of these two factors on metacommunity structuring. We used partial redundancy analysis and Moran's eigenvector maps based on overland and watercourse distances to study the relative importance of environmental control and spatial structuring. We found that, compared with environmental control, spatial structuring was generally negligible, and it did not vary according to our predictions. In general, active dispersers with terrestrial adults showed stronger environmental control than the two passively dispersing groups, suggesting that the species dispersing actively are better able to track environmental variability. There were no clear differences in the results based on watercourse and overland distances. The variability in metacommunity structuring among basins was not related to the differences in the environmental heterogeneity and spatial extent. Our study emphasized that (1) environmental control is prevailing in stream metacommunities, (2) dispersal mode may have an important effect on metacommunity structuring, and (3) some factors other than spatial extent or environmental heterogeneity contributed to the differences among the basins.

## Introduction

The metacommunity concept has been increasingly applied when studying patterns of biodiversity. This concept is based on the notion that both local-scale factors (e.g., local abiotic conditions and interspecific interactions) and large-scale factors (e.g., dispersal and regional climatic conditions) contribute to spatial variation in community structure (Leibold et al. 2004; Holyoak et al. 2005). In the seminal paper by Leibold et al. (2004), four metacommunity perspectives were introduced to account for variation in local community structure: neutral model, species sorting, mass effects, and patch dynamics. The neutral perspective considers the probabilities of species loss (i.e., extinction, emigration) and gain (i.e., speciation, immigration) in structuring a metacommunity (Hubbell 2001). This model describes a situation where a metacommunity is structured by dispersal limitation, speciation, and ecological drift and not by ecological differences among species. The species sorting perspective follows the ideas of the niche theory and environmental filtering (Leibold et al. 2004). However, in species sorting, sufficient dispersal is needed to allow species to track environmental heterogeneity among sites. The other two perspectives – patch dynamics and mass effects – have been recently suggested to be special cases of the species sorting perspective (Winegardner et al. 2012). In patch dynamics, the interacting species differ from each other in being either good competitors or good colonizers within a uniform environment (Leibold et al. 2004). In the mass effects perspective, high dispersal allows species to exist at sites that are normally considered unsuitable for them (Shmida and Wilson 1985).

Typically, estimating the relative importance of environmental and spatial factors in structuring a metacommunity has been used to discriminate among the four perspectives (Cottenie 2005; Heino 2011; Logue et al. 2011). In these studies, a significant and strong relationship with the spatial arrangement of the sites has been interpreted as evidence for the neutral perspective, and a significant and strong relationship with environmental factors has been linked to species sorting. However, under the mass effects perspective, both spatial structuring and environmental control should be important, thus making it difficult to differentiate between species sorting with limiting dispersal and mass effects (Ng et al. 2009). Additionally, the idea that different processes may act simultaneously in a metacommunity has emerged recently, and discriminating among the perspectives has thus been considered challenging (Thompson and Townsend 2006; Logue et al. 2011; Winegardner et al. 2012).

Dispersal is a key factor structuring metacommunities, and it can be measured directly (e.g., Macneale et al. 2005) or indirectly (e.g., Jacobson and Peres-Neto 2010). Because of the difficulties in directly determining dispersal rates and distances in multispecies metacommunities, researchers typically rely on proxies for dispersal (Jacobson and Peres-Neto 2010). Thus, in contemporary metacommunity studies, spatial relationships between sites based on eigenfunction spatial analyses (e.g., Moran's eigenvector maps; see Griffith and Peres-Neto 2006) are commonly used as proxies for dispersal (Logue et al. 2011) or for unmeasured and spatially structured environmental variables (Dray et al. 2012).

Some studies have evaluated the potential effects of dispersal by comparing the metacommunity structure of differently dispersing organisms surveyed at the same set of sites. Some of these studies have assigned species in different groups according to their expected dispersal distances and found that spatial structuring is more evident for species that disperse only short distances compared with species that potentially disperse longer distances (Thompson and Townsend 2006; Astorga et al. 2012). Other studies have compared different taxonomic groups with the expectation that the groups differ in their dispersal ability (Tuomisto et al. 2003; Hájek et al. 2011; Bonada et al. 2012; De Bie et al. 2012). These studies have found, for example, that for passively dispersing species, spatial effects are stronger for organisms with large propagules (e.g., vascular plants, mollusks) than for organisms with small propagules (e.g., diatoms, bryophytes) (Hájek et al. 2011; De Bie et al. 2012), supporting the idea that decreasing propagule size increases the dispersal rates for passively dispersing species (Finlay and Fenchel 2004). However, the importance of dispersal mode *per se* has not been studied extensively (but see Schulz et al. 2012). It can be hypothesized that species that disperse actively should be more able to track environmental variability compared with passively dispersing species of relatively similar size, because passive dispersal should be more stochastic than active dispersal. Among passively dispersing species, on the other hand, species differ in their dispersal strategy (e.g., animalborne, windborne, and waterborne dispersal; Bilton et al. 2001), which may have important implications for the distributions of species (Rundle et al. 2007).

In addition to the dispersal characteristics of the organisms, the spatial connectivity of a system may also affect species distributions. Traditionally, a metacommunity has been viewed in a lattice network where patches are embedded in an unsuitable matrix, but all the patches can be linked to each other via dispersal (Leibold et al. 2004). In dendritic systems, like streams, both the links and patches are more or less suitable habitats, and local habitats lack distinct boundaries (e.g., streams are continuums of shallower riffles and deeper pools; Grant et al. 2007). Different species may differ in their sensitivity to dendritic structure. Species that disperse only via stream corridors (i.e., within-network dispersal) should be more affected by the dendritic nature of the network than those species that are able to disperse overland (i.e., out-of-network dispersal) among streams. The studies that have compared the roles of overland and watercourse distances in explaining community structure have not found very clear differences between the distance measures, except for organisms, such as fish, which are strictly restricted to aquatic habitats (Beisner et al. 2006; Nabout et al. 2009; Landeiro et al. 2011; Maloney and Munguia 2011).

Spatial extent (i.e., mean distance of sampling sites to the centroid of the region) and environmental heterogeneity (i.e., variation in local environmental conditions among the sites) may also have important influences on the relative importance of spatial and environmental processes in shaping community structure. With increasing spatial extent, fewer species are able to disperse across the whole study region, resulting in increased dispersal limitation. Thus, a higher amount of variation in community structure should be explained by spatial variables (Mykrä et al. 2007; Ng et al. 2009; Heino 2011). On the other hand, increasing environmental heterogeneity in a region should lead to stronger environmental filtering and to a higher amount of variation in community composition explained by environmental variables. In other metacommunity studies, the spatial extents have varied several orders of magnitude even for the same group of organisms (Bonada et al. 2012; Göthe et al. 2012; Alahuhta and Heino 2013). However, as an increase in spatial extent is generally associated with an increase in environmental heterogeneity, it has been difficult to evaluate the independent contribution of each factor (e.g., Landeiro et al. 2012).

Recent studies have found that, as a whole, macroinvertebrate communities in headwater streams are mainly structured by local environmental conditions (Brown and Swan 2010; Heino et al. 2012; Siqueira et al. 2012). Here, we expand on previous findings by assigning macroinvertebrates into three dispersal mode groups: passive dispersers with aquatic adults (PaAq), passive dispersers with terrestrial winged adults (PaTe), and active dispersers with terrestrial winged adults (AcTe; note that the abbreviations refer to the first two letters of the words *Pa*ssive, *Ac*tive, *Aq*uatic, and *Te*rrestrial).

We examined the amount of variation in local community composition within a metacommunity that was purely related to environmental variables and purely related to spatial variables. Then, we compared the strength of environmental and spatial signals for the three dispersal mode groups. We had five main predictions. (1) The pure environmental fraction should be higher for active dispersers than passive dispersers, because active dispersers are expected to be better able to track environmental variation than passively dispersing species. (2) The pure spatial fraction should be higher for the group of species that have aquatic adults than for the two groups with terrestrial winged adults due to higher degrees of dispersal limitation. (3) Among the three dispersal mode groups, the following patterns in the strength of pure environmental and spatial processes are expected due to differences in the ability to track environmental heterogeneity. (3a) Environmental control: PaAq < PaTe < AcTe and (3b) Spatial structuring: PaAq > PaTe > AcTe. (4) Watercourse distances will produce better spatial predictors for all dispersal mode groups than overland distances, because most dispersal occurs within stream corridors (e.g., Petersen et al. 2004). However, this pattern is expected to be more visible for PaAq, because species in this group are most strongly restricted to the aquatic environment. (5) Finally, we also expect differences in environmental control and spatial structuring among the three drainage basins. (5a) Environmental control should be the highest in the drainage basin with the highest environmental heterogeneity, and (5b) spatial structuring should be the highest in the study area with the largest spatial extent.

## Materials and Methods

### Datasets, study regions, and macroinvertebrate sampling

We re-analyzed three stream macroinvertebrate datasets, used previously in studying community–environment relationships and species co-occurrence patterns (Heino et al. 2012, 2014; Heino 2013). As these datasets are highly comparable and of high quality (e.g., exactly the same survey protocols, an extensive set of environmental variables and a strict identification level), they are also ideal for this study. Importantly, in these three datasets, environmental heterogeneity and spatial extent are not positively related (Heino 2013), allowing comparisons of these two factors underlying metacommunity structuring.

**Figure 1:**
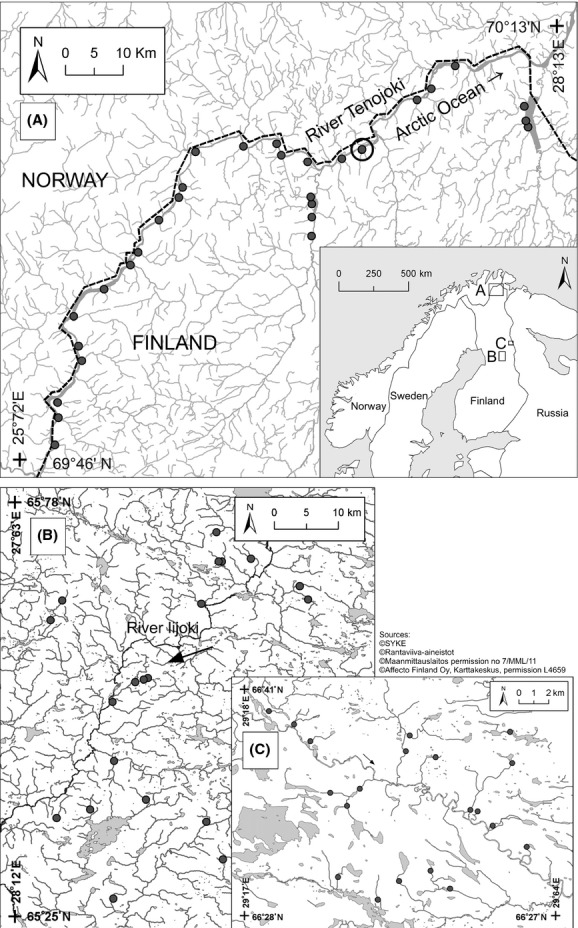
Map of the three study areas (Heino et al. 2014): (A) Tenojoki, (B) Iijoki, and (C) Koutajoki. Note that all the study sites are located in tributaries, although due to the resolution of the map, some sites seem to be located in the main channel of the River Tenojoki. The circle in the uppermost map denotes two sites that are located very close to each other.

The three study regions, Iijoki, Koutajoki, and Tenojoki, are located in northern Finland (Fig. 1, Table 1). From these regions, altogether 70 riffle sites from streams ranging from first to fourth orders were sampled. All sampled sites were near-pristine or pristine. Sampling was conducted in the spring season to facilitate species-level identification. This is the time of the year when most macroinvertebrates are still in the larval stage but close to their maximum size. Because of the short spring season in northern regions and only one field crew available to us, sampling of three drainage basins was not possible in a single year. Thus, the three drainage basins were sampled in consecutive years. The field sampling was conducted soon after the snowmelt, ensuring that the sampling was conducted at a comparable time of the season each year.

**Table 1:** Basic information about the three study areas. Annual precipitation and annual mean temperature are the mean values for the nearest meteorological stations (years 1981–2010; interpolated values based on 10 × 10 km grid data; Finnish Meteorological Institute)

	Iijoki	Koutajoki	Tenojoki
Region's midpoint	65^o^N, 27^o^E	66^o^N, 29^o^E	70^o^N, 27^o^E
Number of sites sampled	20	20	30
Time of sampling	Late May 2009	Late May 2008	Early June 2010
Drainage basin characteristics	Middle boreal coniferous forest and peatlands	Northern boreal coniferous forests; mixed-deciduous; riparian woodlands; nutrient-poor bogs; fertile fens	Arctic-alpine vegetation; mountain birch woodlands at low altitudes; barren fell tundra at higher altitudes
Annual precipitation	721 mm	619 mm	550 mm
Annual mean temperature	0.5°C	−0.5°C	−1.8°C
Area of drainage basin	14,200 km^2^	24,500 km^2^	16,400 km^2^
Area of study region	2150 km^2^	150 km^2^	5370 km^2^

At each site, a kick-net (net mesh size 0.3 mm) sample representing a riffle of approximately 100 m^2^ was taken (for more details, see Heino et al. 2014; and Heino 2013). Four 30-sec per one-meter subsamples divided among the different microhabitats (based on variation in velocity, depth, moss cover, and particle size) were taken and pooled in the field. This sampling effort typically yields more than 70% of species occurring at a site in a given season, mainly missing species that are only occasional in streams (Mykrä et al. 2006). Many previous ecological studies of stream macroinvertebrate communities have omitted mites (Hydracarina), nonbiting midges (Diptera: Chironomidae) and blackflies (Diptera: Simuliidae). These groups, however, typically account for much of stream macroinvertebrate communities in terms of species richness and abundance (Heino 2005). Thus, the lowest possible level of identification was considered important, and all macroinvertebrates, including also midges, blackflies, and mites, were identified to species, species group, or genus level, with the exception of a few individuals of worms that were identified to the family.

### Definition of dispersal mode groups

We used the dispersal mode categorization described by Heino (2013). Thus, macroinvertebrates were assigned into the following three dispersal mode groups (Bilton et al. 2001; Bohonak and Jenkins 2003; Van de Meutter et al. 2007): (1) passive dispersers with aquatic adults (PaAq; i.e., Tricladida, Nematoda, Oligochaeta, Hirudinea, Gastropoda, Bivalvia, Acari, Crustacea), (2) passive dispersers with terrestrial winged adults (PaTe; i.e., Diptera with small body size: Ceratopogonidae, Chironomidae, Simuliidae, Psychodidae, Dixidae, Culicidae), and (3) active dispersers with terrestrial winged adults (AcTe; i.e., Ephemeroptera, Odonata, Plecoptera, Megaloptera, Trichoptera, Coleoptera, Diptera with large body size: Tipuloidea, Empididae, Muscidae). Within the dispersal mode groups, there is probably much among-species variation. However, in general, these groups should differ in their dispersal routes (aerial *versus* watercourse) and the ability to actively search for environmentally suitable sites. The distinction between PaTe and AcTe was primarily based on their body size and assumed ability to resist wind (e.g., Crosskey 1990). However, it can be argued that blackflies (Simuliidae) do disperse actively because they search for blood meals. Thus, preliminary analyses were also conducted with blackflies in the active group to ensure that this would not affect the results much.

### Environmental variables

At each site, 13 riparian, in-stream and water chemistry variables were measured (Table 2). The detailed description of sampling of local environmental variables can be found elsewhere (Heino 2013; Heino et al. 2014). Long-term annual mean temperature, temperature seasonality, and annual precipitation, downloaded from WorldClim database (Hijmans et al. 2005) at the highest resolution (30 arc-seconds, approximately 1 km), were also used in the across-basins analyses described below. This selection of climate variables was made to minimize multicollinearity problems and because these variables are also considered important predictors of biodiversity patterns (e.g., Hawkins et al. 2003).

**Table 2:** Mean, minimum, maximum, and standard deviation (SD) of local environmental variables, species richness, and abundance at local riffle sites in the three drainage basins and in all basins combined. Also, the watercourse and overland distances between sites are shown

	Iijoki basin (n = 20)			Koutajoki basin (n = 20)			Tenojoki basin (n = 30)			All basins (n = 70)		
				
	Mean	Min–max	SD	Mean	Min–max	SD	Mean	Min–max	SD	Mean	Min–max	SD
Conductivity (mS/m)	2.1	1.5–3.1	0.4	7.0	2.8–17.5	3.7	1.8	1.2–2.4	0.3	3.4	1.2–17.5	3.0
pH	6.4	5.7–6.9	0.3	7.3	6.8–7.9	0.3	6.6	6.3–6.7	0.1	6.7	5.7–7.9	0.5
Shading (%)	34	10–70	20	44	5–85	26	16	0–55	14	29	0–85	23
Deciduous (%)	35	5–80	19	44	10–75	15	100	98–100	0	65	5–100	33
Stream width (cm)	304	100–650	131	299	78–1200	266	575	88–2400	506	418	78–2400	388
Depth (cm)	24	16–35	7	25	10–46	10	19	13–33	5	22	10–46	8
Velocity (m/s)	0.4	0.2–0.7	0.2	0.5	0.2–1	0.2	0.4	0.1–0.6	0.1	0.4	0.1–1	0.2
Macrophytes (%)	44	1–78	23	11	0–43	15	4	0–16	4	18	0–78	23
Sand (%)	10	0–49	12	11	0–73	18	1	0–22	5	7	0–73	13
Gravel (%)	6	0–37	9	9	0–30	8	2	0–25	5	5	0–37	8
Pebble (%)	10	0–55	14	33	0–64	19	15	1–65	12	19	0–65	17
Cobble (%)	29	2–53	14	26	0–61	16	45	10–81	20	35	0–81	19
Boulder (%)	44	0–82	25	20	0–92	24	37	1–83	22	34	0–92	25
Local richness												
PaAq	4	0–7	2	3	0–8	2	1	0–3	1	2	0–8	2
PaTe	14	6–25	4	15	7–27	6	10	2–20	4	12	2–27	5
AqTe	18	10–26	5	19	5–31	7	10	2–19	5	15	2–31	7
All taxa	36	22–49	9	36	18–53	10	21	7–40	8	30	7–53	11
Local abundance												
PaAq	22	0–98	25	20	0–115	33	3	0–21	4	13	0–115	24
PaTe	335	36–1045	283	223	10–893	274	60	4–175	47	185	4–1045	240
AqTe	423	70–1685	371	219	47–571	155	247	17–1073	221	289	17–1685	269
All taxa	780	199–1875	473	462	171–1504	383	310	61–1269	253	488	61–1875	410
Watercourse distances (km)	105	0.78–213	59	30	1.54–86	67	57	0.94–165	65	–	–	–
Overland distances (km)	27	0.52–59	52	8.53	0.49–20	46	37	0.10–100	63	250	0.10–541	80

### Spatial variables

Distance matrices were calculated for both overland and watercourse distances using a geographic information system (GIS). The overland distance matrix was calculated using ArcGIS software, Hawth's Analysis Tools extension (distance between points tool) and Euclidean distances. For watercourse distances, Network Analyst extension (origin-destination cost matrix) was used. For this purpose, the stream vector data from The River Network Information System of Finland (1:10,000, Finnish Environment Institute and The National Land Survey of Finland) was used. Streams in the Tenojoki drainage basin were digitized for this study.

Spatial variables were generated through a technique called Moran's eigenvector maps (MEM; formerly called principal coordinates of neighbor matrices, PCNM; Borcard and Legendre 2002; Borcard et al. 2004; Dray et al. 2006). MEMs with high eigenvalues (e.g., the first eigenvectors) represent broad-scale patterns of relationships among sampling sites, whereas those associated with small eigenvalues represent fine-scale patterns (Griffith and Peres-Neto 2006). MEMs represent spatial structures that could be generated by environmental autocorrelation and/or processes such as dispersal (Dray et al. 2006). Following this procedure, spatial variables were generated, considering each basin separately (overland and watercourse distances) and considering all basins together (overland distances only because watercourse distances are not adequate at this scale, as the river basins drain into the Arctic Ocean or the Baltic Sea).

### Ordination, environmental variability, and beta diversity

To reduce the impact of very abundant species, abundance data were log (*Y* + 1)-transformed prior to the analyses described below. As recommended by Legendre and Legendre (2012), the standardized Euclidean and the Bray–Curtis coefficients were used to calculate the distance matrices between streams according to the environmental and biological datasets (for the whole community data and for each dispersal mode group), respectively. Each distance matrix was then submitted to a principal coordinate analysis (PCoA) to visualize the main patterns of similarity among the streams according to each dataset. A permutational multivariate analysis of variance (NPMANOVA; Anderson 2001) was run to test for differences in community structure and environmental characteristics among the basins. Then, an analysis of multivariate homogeneity of group dispersions (PERMDISP; Anderson 2006) was conducted with the goal of testing for differences in multivariate dispersions among streams localized in different basins. We applied PERMDISP to a “species-by-streams” data table. Thus, PERMDISP tests the null hypothesis of no difference in beta diversity among drainage basins (see Anderson et al. 2006).

To ensure that the environmental variability and spatial extent were not related in our datasets, we used a subsampling approach. From each of the basins, we subsampled datasets of eight sites located within an extent that was equal to the extent in the smallest study area (the Koutajoki basin). Then, following the same analyzing protocol as above, we analyzed the environmental heterogeneity within these subsampled datasets while keeping the spatial extent constant.

### Variation partitioning

When both species and the environment are spatially structured, it is necessary to filter out the effects of spatial correlation when testing for the importance of ecological factors, such as environmental predictors, to avoid inflated Type I error rates (Peres-Neto and Legendre 2010). Here, this was performed using a variation partitioning procedure (Borcard et al. 1992) applied to the RDA models (partial RDA). This analysis estimates the percentage of variation in the species data that could be attributed exclusively to different fractions: total explained variation [a + b + c], environmental variation [a + b], spatial variation [b + c], environmental variation without the spatial fraction [a], spatial variation without the environmental fraction [c], the common fraction of variation [b] shared by environmental (**E**) and spatial predictors (**S**), and the residual fraction of variation not explained by **E** and **S** [d] (Peres-Neto et al. 2006). The partial RDAs were run for each dispersal mode group and for the whole community in each basin separately and for all basins together.

We partitioned the variance in response datasets (i.e., species data tables of each dispersal mode group) into fractions explained by explanatory datasets (i.e., environmental data and spatial filters). Species data tables were Hellinger-transformed (Legendre and Gallagher 2001) prior to variation partitioning, because this transformation makes community composition data containing many zeros more suitable for statistical methods with linearity assumptions, such as redundancy analysis (RDA).

### Comparison of stream distance measures

The comparison of the distance measures (overland vs. watercourse) was conducted using the spatial fraction [b + c]. This was carried out because the distance measures may differ in their ability to represent environmental variables, and thus, the varying shared fraction [b] could lead to confounded pure fractions [c].

### Comparison of dispersal mode groups

To enable the comparisons between the dispersal mode groups and the drainage basins, we used a priori selected sets of environmental variables in partial RDA. Thus, based on previous studies on macroinvertebrate communities in northern streams (Mykrä et al. 2007; Heino et al. 2012, 2014), we selected pH, conductivity, width, and macrophyte cover. In addition, we used annual mean temperature, temperature seasonality, and annual precipitation when analyzing the across-basins data. All MEM variables (eigenvectors) with significant patterns of spatial autocorrelation, that is, with positive and significant Moran's *I* (*P* < 0.05; see Sokal and Oden 1978a,b), were used in partial RDA.

Differing species richness and patterns of rarity and commonness may lead to different levels of information content in the species matrices. This, in turn, is likely to affect the levels of adjusted R^2^ in variation partitioning (Lennon et al. 2004; Siqueira et al. 2012). Thus, for each species data table, we calculated the information content IC = ∑P_i_ × (1–P_i_), where P_i_ is the proportion of sites occupied by the i^th^ species (Lennon et al. 2004). Then, we created matrices of all dispersal mode groups containing the same amount of information content than the group with lowest information content. This was carried out within each basin by randomly sampling species in dispersal mode group matrices with higher information content to obtain datasets (response matrices) with the same information content (± 0.03). We created 999 response matrices in each case and re-run the partial RDA. Then, we calculated the mean and standard deviation (SD) of the fractions.

All results were based on adjusted fractions of variation (Peres-Neto et al. 2006). Analyses were performed in the R environment (R Core Team 2013) using vegan (Oksanen et al. 2013).

## Results

### Spatial variables

The number of MEMs with positive and significant Moran's *I* coefficients when using watercourse distances was two for the Iijoki and Koutajoki basins and five in the Tenojoki basin. When using overland distances, the numbers of MEMs with positive and significant Moran's *I* were 4, 5, and 7, respectively. When all the basins were analyzed together (conducted only for overland distances), only one MEM was obtained.

### Environmental variability

**Figure 2:**
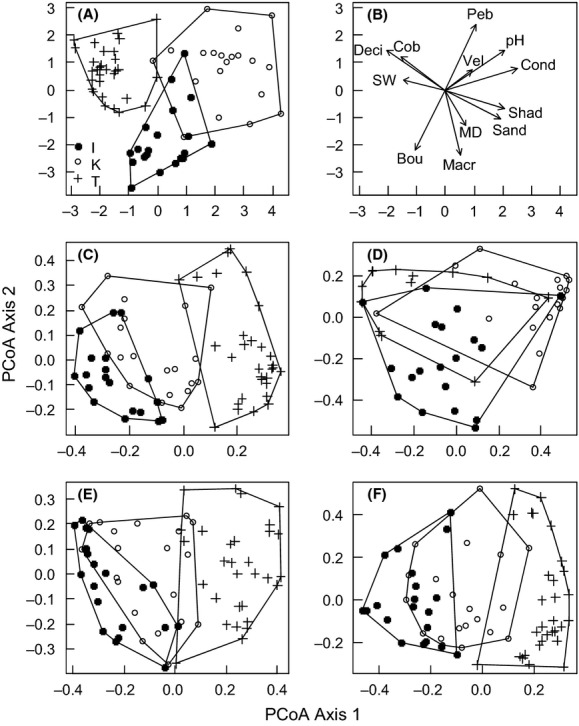
Scores derived from a PCoA applied to the environmental data (A) and the correlation coefficients between the variables and these scores (B). Scores derived from a PCoA applied to the whole biological data (C), passively dispersing species with aquatic adults, PaAq (D), passively dispersing species with terrestrial adults, PaTe (E), and actively dispersing species with terrestrial adults, AcTe (F). I = Iijoki basin, K = Koutajoki basin, T = Tenojoki basin. Environmental variables are referred to using abbreviations of three to four-first letters of the respective variable (conductivity, deciduous tree, macrophytes, shading, velocity, sand, pebble, cobble, boulder), SW = stream width, MD = mean depth.

The three basins were clearly different regarding environmental characteristics (NPMANOVA *F*_2,67_ = 19.9; *P* < 0.01; Fig. 2A, Table 2). In the Tenojoki basin, riparian tree composition was virtually totally dominated by deciduous trees, and streams in this basin, predominantly with cobble substrates, were wider than the streams of the Iijoki and Koutajoki basins (Fig. 2B, Table 2). Streams in the Koutajoki basin, with a high frequency of fine and intermediate sized substrates, were more shaded and characterized by higher ionic concentrations and pH values. Finally, high aquatic macrophyte cover (up to 78%, Table 2) was a distinctive feature of the streams in the Iijoki basin. Besides differences in positions across the multidimensional space, we also detected differences in multivariate dispersions among the basins (PERMDISP; *F*_2,67_ = 7.1, *P* < 0.001, Fig. S2), with the Koutajoki basin being the basin with the highest environmental variability among streams, despite having the smallest spatial extent encompassing the sampled sites. The Tenojoki basin, despite having the largest spatial extent, had the lowest environmental variability among streams. Even when sets of sites with the same spatial extent were subsampled from each basin, the Koutajoki basin had the highest environmental heterogeneity (Fig. S2).

### Biological variability

A total of 228 species were identified from all the three basins (Tables S1 and S2). The total species richness was highest in the Koutajoki basin (159 species) and lowest in the Tenojoki basin (98 species). Of the three dispersal mode groups, PaAq had the lowest total richness. Also, the riffle scale species richness and abundance were lowest for PaAq and, in general, in the Tenojoki basin (Table 2). Proportions of singletons varied between 0% and 29% in a dataset, but none of the basins or none of the dispersal mode groups differed notably from the others in respect to the proportion of singletons (Table S1).

There were significant differences among the three drainage basins in the structure of the whole community (NPMANOVA; *F*_2,67_ = 11.0, *P* < 0.01; Fig. 2C) and all the dispersal mode groups (NPMANOVA; PaAq, *F*_2,57_ = 11.2; PaTe, *F*_2,67_ = 8.11; AcTe, *F*_2,67_ = 12.4; *P* < 0.01; PCoA, Fig. 2D–F). The null hypothesis of homogeneity in the multivariate dispersions among the three basins for the whole community data was rejected (PERMDISP; *F*_2,67_ = 4.4, *P* < 0.05). Again, despite the low spatial extent, higher variation in the species composition of the whole community was detected among streams of the Koutajoki basin than among the streams of the other two basins (Fig. 2C and Fig. S1). Significant among-basin differences in the levels of beta diversity were also detected for AcTe (PERMDISP; *F*_2,67_ = 7.0, *P* < 0.05; Fig. S1). Conversely, no significant differences in beta diversity were detected for PaAq (*F*_2,57_ = 1.6, *P* = 0.190) or PaTe (*F*_2,67_ = 2.2, *P* = 0.122). These results indicate that AcTe largely drove the patterns detected for the whole community (Fig. 2C). In short, we found significant differences in the community structure among drainage basins for the whole community and for the different dispersal mode groups. However, differences in the levels of beta diversity within basins were found only for the whole community and for AcTe.

### Relative importance of environmental and spatial factors

**Figure 3:**
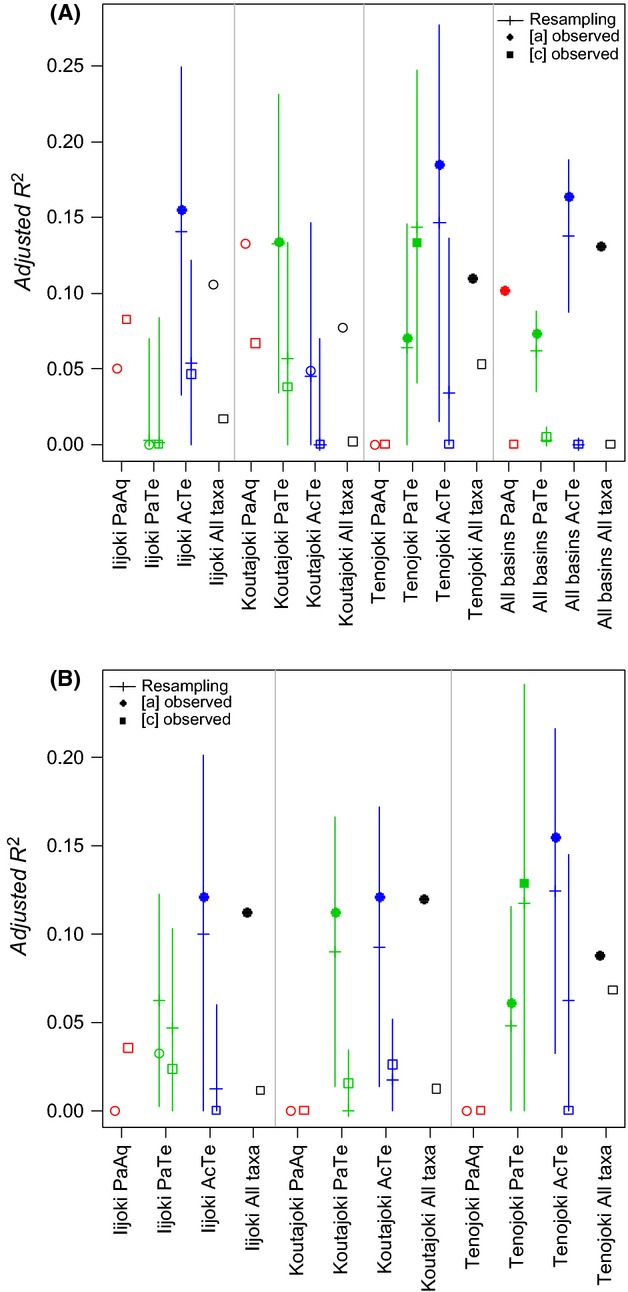
Pure environmental [a] and spatial [c] fractions in variation partitioning when using overland distances (A) and watercourse distances (B). Different colors denote each dispersal mode group to facilitate comparisons between basins and within the dispersal mode groups. Filled symbols indicate significant fractions. Whiskers show mean and standard deviations when the information content of PaTe and AcTe matrices was reduced to the same level as in the most species poor group (PaAq). Negative fractions are converted to 0.

The pure environmental fraction [a] was significant in more than a half of the cases studied (Fig. 3, Table S5 and S6). The amount of variation explained by the significant and pure environmental fractions varied between 6.1 and 18.8%. On the other hand, the pure spatial fraction was significant in only one case, that is, for PaTe in the Tenojoki basin (13% of the variation explained by the spatial variables).

Within basins, AcTe showed the highest pure environmental fractions compared with the other dispersal mode groups in all but one case. Only in the Koutajoki basin when using overland distances, the pure environmental fraction was not significant for AcTe, but significant and the highest for PaTe (Fig. 3, Table S5). Thus, within the basins, the results partly supported the predictions 1 and 3a (Table 3). Because spatial fraction was not significant in almost all the cases, we conclude that the results did not support the predictions 2 and 3b (Table 3).

**Table 3:** A priori predictions, description of the results based on the analyses, and their interpretation. PaAq = actively dispersing species with aquatic adults, PaTe = passively dispersing species with terrestrial adults, AcTe = actively dispersing species with terrestrial adults, K = Koutajoki basin, I = Iijoki basin, and T = Tenojoki basin

Prediction	Result	Interpretation
(1) Pure environmental component is higher for active dispersers.		
Within basins	PARTIAL SUPPORT (fraction highest except in Koutajoki when using overland distances)	Suggests that, at small spatial extents, the actively dispersing species are more able to track environmental heterogeneity than passively dispersing species.
Across basins	SUPPORTED	Suggests that, at the large spatial extent, actively dispersing species are more able to track environmental heterogeneity than passively dispersing species.
(2) Pure spatial component is higher for species with aquatic adults.		
Within basins	NOT SUPPORTED	In this system, in general, the importance of spatial structuring is so low that it hinders meaningful comparisons between dispersal mode groups.
Across basins	NOT SUPPORTED	See above.
(3a) Strength of pure environmental control: AcTe > PaTe > PaAq		
Within basins	PARTIAL SUPPORT (In Koutajoki with overland distances, PaTe had the highest fraction)	See prediction 1.
Across basins	NOT SUPPORTED	Due to high covariation, the effects of spatial location and environmental factors cannot be distinguished at large spatial extent.
(3b) Strength of pure spatial control: PaAq > PaTe > AcTe		
Within basins	NOT SUPPORTED	See prediction 2.
Across basins	NOT SUPPORTED	See prediction 2.
(4) Spatial [b + c] fraction should be higher when using watercourse distances compared with overland distances.	NOT SUPPORTED	The two distance measures are equally good or poor in the studied system.
(5a) Environmental structuring should be highest in the drainage basin with the highest environmental heterogeneity (K > I > T)	NOT SUPPORTED	Some factor(s) other than the degree of environmental heterogeneity drove the differences among basins.
(5b) Spatial structuring should be highest in the study area with the largest spatial extent (T > I > K)	NOT SUPPORTED	Some factor(s) other than the spatial extent drove the differences among basins.

When partial RDA was conducted across all the basins (using overland distances only), the results revealed significant pure environmental fractions, nonsignificant pure spatial fractions, and high shared fractions for all dispersal mode groups. The pure environmental fraction was the highest for AcTe (16%) and the lowest for PaTe (7%). Thus, across basins, the results supported the prediction 1 but did not support the more detailed prediction 3a (Table 3).

We did not find any consistent patterns when comparing the fractions among basins within each dispersal mode group. The most environmentally heterogeneous basin, that is, the Koutajoki basin, had the highest environmental fraction for PaTe (using both distance measures) and for the whole community (watercourse distances; Fig. 3, Tables S5, S6). For AcTe, the highest pure environmental fraction was found in the least environmentally heterogeneous basin, Tenojoki (using both distance measures). Also, when using overland distances, the whole community had the highest pure environmental fraction in Tenojoki basin. Thus, we did not find support for the prediction 5a. Because of the high number of nonsignificant pure spatial fractions, we neither found support for prediction 5b (Table 3).

Our findings remained largely the same regardless of the distance metric (i.e., watercourse versus overland; Fig. 3, Tables S5 and S6). The [b + c] fraction was used to compare the amount of variation that the two distance metrics were able to explain in each case. Throughout the results, the differences were small. For instance, the highest difference between the two types of distance measures was 0.04 (AcTe in the Koutajoki basin; Table S4). Thus, the results did not support prediction 4.

The conclusions related to the predictions one to three did not change when the information content of PaTe and AcTe matrices was reduced to the same level as in the most species poor matrices of PaAq (Fig. 3, Tables S3–S6). Mean values of each set of the resamplings were in most cases close to the observed values. In the cases where the mean differed from the observed value, the conclusions did not change. In the cases where observed fractions were significant, the mean of the resampled data was often lower as observed adjusted R^2^.

## Discussion

### Effects of dispersal mode

We found that the importance of environmental control was almost always stronger than that of spatial structuring, supporting the idea of “power of species sorting” (Cottenie 2005; Van der Gucht et al. 2007). In general, the actively dispersing group with terrestrial adults (AcTe) had stronger environmental relationships compared with the passively dispersing species (prediction 1, Table 3). This finding suggests that actively dispersing species are possibly better able to track environmental variability than passively dispersing species. Previous studies have emphasized the effect of potential dispersal distances of species on community structuring (e.g., Astorga et al. 2012), but our results suggest that, in addition to the dispersal distances, also the dispersal mode may affect the structuring of metacommunities (but see Schulz et al. 2012). However, although there is evidence that aquatic insects are able to actively select their preferred habitats (e.g., Vonesh et al. 2009), information on their ability to actively search for suitable habitats and direct their flight toward such habitats is still lacking. We thus encourage researchers to compare other organism groups that differ in their dispersal strategies but are of relatively similar size (e.g., various groups of insects).

The passively dispersing group with terrestrial adults (PaTe) was the only group showing a significant spatial fraction within a basin (i.e., the Tenojoki basin). It is unlikely that the spatial signature of PaTe would be caused by dispersal limitation in a system where less effectively dispersing species (PaAq) did not show significant spatial signature. A spatial signal may also appear due to an effect of excessive dispersal (Cottenie 2005; Ng et al. 2009). Mass effects (i.e., the presence of species in environmentally suboptimal sites due to intense dispersal from environmentally suitable sites) may obscure community–environment relationships, as dispersal from a source-neighboring site allows persistence at a sink site, resulting in a significant spatial signal in variation partitioning. It can be speculated that PaTe, which may sometimes disperse in very high numbers and across large distances (e.g., Johnson 1969), could be subjected to mass effects, at least in some parts of the basin.

By contrast, AcTe and PaAq did not have a significant spatial signature in any of the basins or across the basins. For AcTe, this finding suggests that species in this group do not show clear signs of dispersal limitation. Indeed, previous studies have found little spatial structuring of organisms belonging to this group (e.g., mayflies, stoneflies, and caddisflies), although most of these studies have been conducted over smaller spatial extents than our present study (Heino and Mykrä 2008; Landeiro et al. 2012). Conversely, it is not easy to explain the lack of spatial structuring for PaAq, as species in this group were expected to be dispersal limited with a strong spatial signal. The explanation could be that although PaAq cannot actively select suitable environment, many species in this group are distributed by other actively dispersing organisms, such as waterfowl, aquatic insects, and amphibians (Bilton et al. 2001). Thus, it might be that this dispersal strategy is enough to prevent strong dispersal limitation. These unexpected results for PaAq could also be due to the lower species richness and abundances of PaAq compared with other two dispersal mode groups. However, when the information content of species data tables was reduced to the same level in all the groups, the conclusions remained the same, thus indicating the robustness of our findings.

The very low spatial fraction in the across-basin analyses suggests that dispersal limitation is not important for stream invertebrates even at such large spatial extents. Instead, the large shared fraction reflects the fact that environmental variables are spatially structured. Indeed, the climatic gradient (and also the gradient for some local environmental variables) is the most prominent along the south-north axis, which is also the main geographical gradient in our data at the large spatial extent. As noted previously elsewhere (Gilbert and Bennett 2010; Smith and Lundholm 2010), it is sometimes difficult to distinguish between environmental constraints and potential dispersal limitation using variation partitioning.

It is never straightforward to categorize all species in multispecies assemblages to coarse classes based on biological traits. Also, the dispersal mode categorization we used can be criticized. For example, in some studies, all insect species, including midges that were assigned to PaTe in our study, have been considered as active (e.g., De Bie et al. 2012). However, as the PaTe species are generally small in size and rather weak fliers, their movement is strongly affected by winds (Johnson 1969; Rundle et al. 2007). Thus, we believe that especially the long-distance dispersal of these species is more passive than active and, hence, at the spatial scale we studied (the pairwise distances within a basin varying from 0.1 to 100 km), it is reasonable to call these species passive. On the contrary, the species of AcTe are generally larger in size and stronger fliers, and hence, they should be more able to direct their flight (e.g., Rundle et al. 2007) and locate environmentally suitable habitats (Heino 2013).

### Comparison of overland and watercourse distances

Overland and watercourse distance measures were equally poor descriptors of community structure of each dispersal mode group in the within-basin analyses (prediction 4, Fig 3, Tables S4–S6). The differences between the two spatial distance measures might be more visible in drainage basins, where spatial processes are more important than environmental structuring. Additionally, as all the riffle sites sampled in our study were located in different streams, the potential dispersal routes via watercourses also included sections of large rivers, which may have decreased the use of stream corridors compared with the importance of overland dispersal. However, previous studies on stream macroinvertebrates have not found clear differences between overland and watercourse distances (e.g., Landeiro et al. 2011), or overland distances have actually explained variation in community structure better than watercourse distances (e.g., Maloney and Munguia 2011). Thus, despite the findings that stream macroinvertebrates often prefer stream corridors for dispersal (e.g., Petersen et al. 2004), they may also disperse efficiently over land.

### Spatial extent, environmental heterogeneity, and metacommunity structuring

Spatial extent and environmental heterogeneity of a study region may influence metacommunity structuring. For example, Heino et al. (2012) found that the metacommunity structuring of algal, bryophyte, and macroinvertebrate communities was not similar in two boreal drainage basins (the Iijoki and Koutajoki basins). Two possible reasons for differences between these basins were indeed differences in environmental heterogeneity and spatial extent. Here, we re-analyzed the stream macroinvertebrate data and collected an additional dataset with larger sampling extent, but from an area which was assumed to have lower environmental heterogeneity (the Tenojoki basin). Thus, unlike in many datasets (e.g., Landeiro et al. 2012), environmental heterogeneity and spatial extent were not positively related in our data, which was also ensured by subsampling all the datasets based on the same spatial extent. The unrelatedness of spatial extent and environmental heterogeneity allowed comparisons of these two underlying factors in structuring macroinvertebrate metacommunities. Although we found some variability in the processes structuring metacommunities of the dispersal mode groups among the basins, which were evidenced by RDA, PCoA, PERMANOVA, and PERMDISP, this variability was not related to the differences in environmental heterogeneity and spatial extent of the regions.

Landeiro et al. (2012) also compared three different-sized regions and did not find support for the prediction that the spatial structuring of a caddisfly metacommunity should increase with increasing spatial extent. Other explanations for among-region variation in metacommunity structuring may be related to differences in landscape characteristics that affect matrix permeability (e.g., Biswas and Wagner 2012) or harshness of environmental conditions. Thus, although the northernmost basin in our study (the Tenojoki basin) had the lowest level of environmental variability, the harsh winter and spring conditions in this basin may have increased the relative importance of environmental structuring compared with that in the two more southerly drainage basins (the Iijoki and Koutajoki basins). On the other hand, the among-basin differences in community composition might be explained by the differences in the species pools among the basins. Thus, biogeographical and historical factors may override the effects of environmental heterogeneity and spatial extent on metacommunity structuring. Unfortunately, the three ideas above remain largely speculative, because the limited number of regions hinders formal statistical testing.

Using the same dataset of the three drainage basins, Heino (2013) found that both environmental heterogeneity and dispersal mode affected the patterns of co-occurrence of stream macroinvertebrate species pairs within each basin. Furthermore, Heino et al. (2014) found that there were no clearly distinct community types but rather the community variation was continuous along environmental gradients across the three basins. These two studies also exemplify that spatial effects are probably minor at the spatial scales studied and that species sorting is prevailing in these systems. Thus, although the details of community–environment relationships do differ among the drainage basins (Heino et al. 2012), the environment is clearly superior to dispersal limitation in affecting stream metacommunities irrespective of the dispersal mode of the organisms.

## Conclusions

We found some differences among the dispersal mode groups, suggesting that in addition to dispersal distances, dispersal mode also affects metacommunity structuring. In general, the group of actively dispersing species with terrestrial adults showed stronger environmental control than the two passively dispersing groups. This finding suggests that actively dispersing species are better able to track environmental heterogeneity than passively dispersing species. Although environmental heterogeneity and spatial extent were unrelated, neither of these factors seemed to explain the differences among the basins. For each dispersal mode group, spatial structuring was generally negligible in comparison with environmental control, suggesting that species sorting is prevailing in stream metacommunities, even for groups of species with varying dispersal strategies.

## Supporting Information

**Figure S1.** Average distances to centroid for each dispersal mode group and the whole community data.

**Figure S2.** Environmental heterogeneity in the three study areas.

**Table S1.** Total numbers of taxa and proportion of singletons in each dispersal mode group and in each basin.

**Table S2.** A list of taxa detected in the field surveys (with frequency of occupancy and mean abundance). The taxa are grouped by study region and dispersal mode group.

**Table S3.** Number of species and information content within each dataset.

**Table S4.** Adjusted R^2^ values and p-values for environmental [a+b] and spatial [b+c] models.

**Table S5.** Pure environmental and pure spatial fractions for observed data, as well as when information content was taken into account (based on overland distances).

**Table S6.** Pure environmental and pure spatial fractions for observed data and when information content was taken into account (based on watercourse distances).
