# Supplementary material for: Metacommunity structuring in stream networks: roles of dispersal mode, distance type, and regional environmental context
Source: Ecol Evol. 2013 Oct 14;3(13):4473–87. doi: 10.1002/ece3.834 (PMC3856747; doi:10.1002/ece3.834)
Supplement: Supplementary file 1 [file ece30003-4473-SD1.pdf]

Online supporting information

Metacommunity structuring in stream networks: roles of dispersal mode, distance type and regional environmental context

M. Grönroos, J. Heino, T. Siqueira, V.L. Landeiro, J. Kotanen & L.M. Bini

Fig. S1. Average distances to centroid for each dispersal mode group and whole biological data. Different letters indicate groups that are significantly different (P < 0.05). Pairwise comparisons were not made if the overall PERMDISP test was not significant. I = Iijoki basin, K = Koutajoki basin, T = Tenojoki basin.

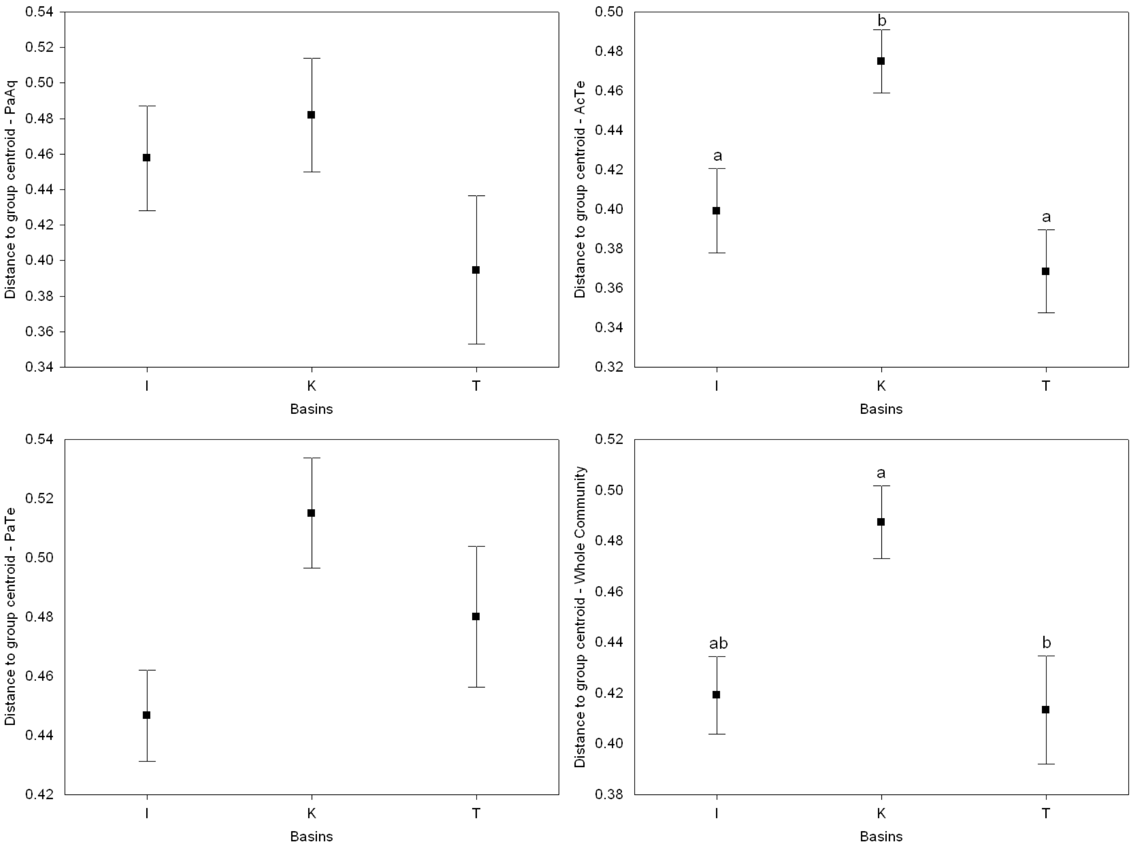

Online supporting information

Metacommunity structuring in stream networks: roles of dispersal mode, distance type and regional environmental context
M. Grönroos, J. Heino, T. Siqueira, V.L. Landeiro, J. Kotanen & L.M. Bini

Fig. S2. Environmental heterogeneity (measured as an average distance to centroid) for subsampled sets of sites (empty symbols) for the three study basins. In the subsampling, eight sites located within an extent that was equal to the extent in the smallest study area (the Koutajoki basin) were sampled. In the Koutajoki basin, eight sets of sites were subsampled. In the Iijoki and Tenojoki basins, there were six and three possible sets of sites, respectively. Also the environmental heterogeneity for all the sites in the given dataset is shown (red filled symbols).

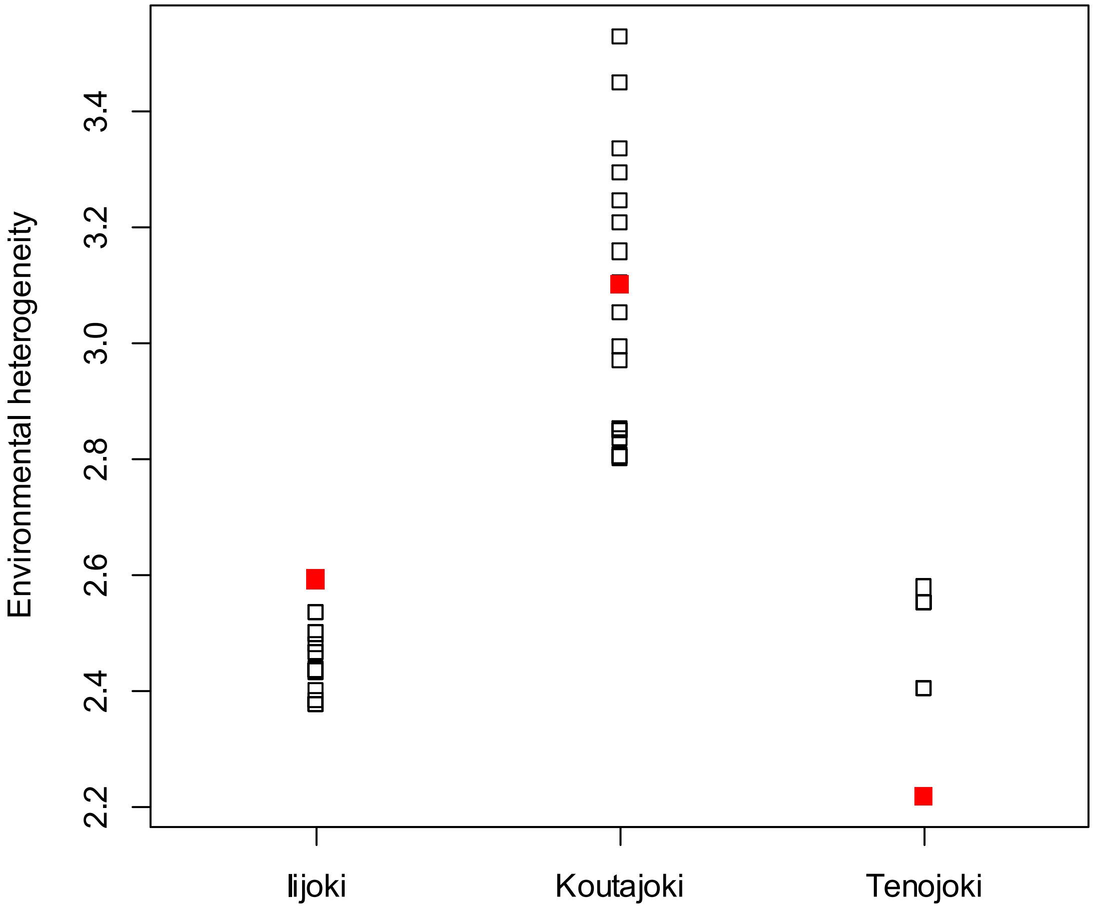

Online supporting information

Metacommunity structuring in stream networks: roles of dispersal mode, distance type and regional environmental context
M. Grönroos, J. Heino, T. Siqueira, V.L. Landeiro, J. Kotanen & L.M. Bini

Table S1. Total number of taxa detected in each dispersal mode group and in each basin. Proportion of singletons (i.e. species that had only one individual within a basin) in parentheses.

	Number of taxa			
Basin	PaAq	PaTe	AcTe	All groups
Iijoki	14 (14%)	64 (17%)	63 (17%)	141
Koutajoki	16 (25%)	75 (17%)	68 (0%)	159
Tenojoki	7 (29%)	52 (8%)	39 (26%)	98
All basins	23	112	93	228

Online supporting information

Metacommunity structuring in stream networks: roles of dispersal mode, distance type and regional environmental context

M. Grönroos, J. Heino, T. Siqueira, V.L. Landeiro, J. Kotanen & L.M. Bini

Table S2. Species list for the three study regions. Frequency (number of sites occupied) and mean abundance at occupied sites are given. Species are grouped into the three dispersal mode groups. Within orders (and within families for the Diptera) species are in alphabetical order to facilitate reading.

	Iijoki basin		Koutajoki basin		Tenojoki basin	
	Frequency	Mean abundance	Frequency	Mean abundance	Frequency	Mean abundance
**<u>Passively dispersing species with aquatic adults (PaAq)</u>**						
**Tricladida**						
Dendrocoelidae sp.	0	0	1	1	0	0
Planariidae sp.	0	0	1	2	0	0
**Nematoda**						
Merminthidae sp.	1	1	2	2	0	0
**Oligochaeta**						
Eiseniella tetraedra	4	3	13	4	2	2
Oligochaeta sp.	10	2	15	4	4	3
**Hirudinea**						
Glossiphonia complanata	0	0	1	2	0	0
**Gastropoda**						
Gyraulus sp.	0	0	5	9	0	0
Radix peregra	0	0	5	3	1	1
Valvata piscinalis	0	0	1	10	0	0
**Bivalvia**						
Pisidium sp.	2	1	7	25	0	0
Sphaerium sp.	3	28	0	0	0	0
Margaritifera margaritifera	1	1	0	0	0	0
**Hydracarina**						
Hydrodroma sp.	2	1	0	0	0	0
Hygrobates longipalpis	9	11	0	0	0	0
Hygrobates nigromaculatus	5	1	0	0	1	1
Hygrobates norvegicus	4	8	0	0	0	0
Hygrobates trigonicus	0	0	0	0	2	1
Lebertia sp.	7	2	1	1	2	1
Limnochares aquatica	2	1	1	1	0	0
Sperchon sp.	16	7	3	2	19	3
**Crustacea**						
Asellus aquaticus	9	4	2	5	0	0
Candona sp.	0	0	1	1	0	0
Gammarus lacustris	0	0	1	9	0	0
**<u>Mainly passively dispersing species with terrestrial adults (PaTe)</u>**						
**Diptera: Psychodidae**						
Berdeniella sp.	5	2	15	8	6	5

Online supporting information

Metacommunity structuring in stream networks: roles of dispersal mode, distance type and regional environmental context

M. Grönroos, J. Heino, T. Siqueira, V.L. Landeiro, J. Kotanen & L.M. Bini

**Diptera: Culicidae**						
Aedes sp.	0	0	4	3	0	0
Culex sp.	3	2	0	0	0	0
**Diptera: Dixidae**						
Dixa submaculata	0	0	1	1	0	0
**Diptera: Ceratopogonidae**						
Bezzia sp.	8	2	11	3	2	3
**Diptera: Chironomidae**						
Apsectrotanypus trifascipennis	0	0	1	1	0	0
Brillia bifida	1	2	6	4	6	2
Brillia longifurca	1	1	1	1	0	0
Bryophaenocladius sp.	0	0	1	1	0	0
Cardiocladius capucinus	0	0	0	0	2	1
Chaetocladius piger	0	0	1	2	1	44
Chaetocladius suecicus	0	0	1	1	0	0
Constempellina brevicosta	0	0	0	0	1	1
Corynoneura celtica	0	0	0	0	3	6
Corynoneura lobata	0	0	0	0	5	1
Corynoneura sp.	9	4	3	1	0	0
Cricotopus pulchripes	0	0	0	0	1	4
Cricotopus annularius	1	1	0	0	0	0
Cricotopus festivellus	4	17	0	0	0	0
Cricotopus laricomalis	3	3	0	0	0	0
Cricotopus sp.	0	0	7	2	0	0
Diamesa bertrami	0	0	0	0	1	3
Diamesa incallida	0	0	0	0	1	1
Diamesa insignipes	0	0	1	2	0	0
Diplocladius cultriger	0	0	2	5	0	0
Epoicocladius ephemerae	0	0	1	4	0	0
Eukiefferiella boevrensis	0	0	0	0	1	4
Eukiefferiella brevicalcar	13	10	6	18	2	15
Eukiefferiella claripennis	6	3	6	5	2	1
Eukiefferiella devonica	10	25	6	18	8	6
Eukiefferiella minor	1	1	2	178	2	6
Heleniella ornaticollis	0	0	2	2	2	2
Heterotanytarsus apicalis	3	1	1	5	0	0
Heterotrissocladius marcidus	2	3	2	3	0	0
Krenosmittia boreoalpina	0	0	0	0	1	3
Lasiodiamesa sp.	0	0	1	1	0	0
Limnophyes sp.	2	2	0	0	0	0
Macropelopia sp.	1	1	1	1	0	0
Metriocnemus terrester	0	0	1	5	0	0
Metriocnemus fuscipes	1	1	0	0	0	0
Micropsectra atrofasciata	0	0	12	7	21	10
Micropsectra junci	9	29	6	5	0	0
Micropsectra logani	0	0	1	7	0	0

Online supporting information

Metacommunity structuring in stream networks: roles of dispersal mode, distance type and regional environmental context
M. Grönroos, J. Heino, T. Siqueira, V.L. Landeiro, J. Kotanen & L.M. Bini

Micropsectra notescens	0	0	1	10	0	0
Micropsectra pallidula	5	17	12	5	0	0
Micropsectra recurvata	0	0	1	8	0	0
Microtendipes pedellus	0	0	2	3	0	0
Nanocladius balticus	1	1	0	0	0	0
Nanocladius rectinervis	8	4	2	4	1	1
Natarsia punctata	0	0	1	2	0	0
Odontomesa fulva	1	2	0	0	0	0
Orthocladius excavatus	0	0	0	0	1	15
Orthocladius frigidus	1	2	1	26	1	2
Orthocladius holsatus	0	0	3	2	0	0
Orthocladius lignicola	4	1	2	1	1	2
Orthocladius olivaceus	0	0	0	0	1	3
Orthocladius rhyacobius	0	0	4	2	3	2
Orthocladius rivicola	0	0	0	0	21	7
Orthocladius saxosus	0	0	1	87	1	2
Paracladopelma laminatum	0	0	1	1	0	0
Parakiefferiella bathophila	0	0	0	0	1	6
Parametriocnemus stylatus	2	2	8	6	0	0
Paraphaenocladius sp.	1	1	0	0	0	0
Paratrichocladius rufiventris	0	0	2	3	1	2
Paratrichocladius skirwithensis	0	0	1	8	9	4
Pentaneurella katterjokki	0	0	0	0	3	2
Polypedilum breviantennatum	2	1	3	1	0	0
Polypedilum convictum	3	2	0	0	0	0
Potthastia longimanus	11	2	7	7	8	2
Prodiamesa olivacea	0	0	1	1	0	0
Psectrocladius psilopterus	1	4	0	0	0	0
Pseudodiamesa branickii	1	1	0	0	1	3
Pseudosmittia gracilis	0	0	1	31	2	3
Rheocricotopus atripes	1	1	1	1	0	0
Rheocricotopus effusus	1	2	2	3	0	0
Rheocricotopus fuscipes	7	22	5	7	0	0
Rheopelopia sp.	6	9	7	13	16	5
Rheotanytarsus sp.	9	4	5	8	1	5
Stempellina bausei	1	1	1	1	0	0
Stempellinella brevis	8	5	2	3	0	0
Stempellinella edwardsi	0	0	0	0	1	2
Stenochironomus sp.	0	0	2	1	0	0
Syndiamesa sp.	0	0	1	4	0	0
Synorthocladius semivirens	2	5	4	6	0	0
Tanytarsus curticornis	0	0	1	1	0	0
Thienemanniella clavicorni	1	2	0	0	0	0
Thienemanniella majuscula	0	0	0	0	6	2
Thienemanniella vittata	8	5	3	2	0	0
Thienemannimyia sp.	10	13	11	13	2	1

Tokunagaia sp.	1	1	1	3	7	3
Trichotanypus posticalis	0	0	0	0	1	1
Trissopelopia longimana	10	10	6	7	11	1
Tvetenia bavarica	2	26	6	9	11	7
Tvetenia calvescens	14	13	13	8	16	4
Tvetenia discoloripes	2	31	8	1	15	6
Zavrelimyia sp.	2	2	4	2	0	0
**Diptera: Simulidae**						
Greniera ivanovae	1	4	0	0	0	0
Helodon ferrugineus	1	3	0	0	0	0
Prosimulium hirtipes	15	37	8	27	18	8
Prosimulium macropyga	2	44	1	93	7	3
Simulium aureum	0	0	1	1	0	0
Simulium beltukovae	2	3	2	5	0	0
Simulium equinum	1	115	0	0	0	0
Simulium lundstromi	1	7	1	6	0	0
Simulium monticola	3	47	3	12	23	15
Simulium murmanum	3	10	0	0	8	6
Simulium noelleri	1	2	0	0	0	0
Simulium ornatum	10	15	13	25	9	2
Simulium paramorsitans	1	4	0	0	0	0
Simulium tuberosum	2	1	0	0	0	0
Simulium vernum	20	173	17	92	19	9
Stegopterna trigonium	3	97	3	104	2	3
**Mainly actively dispersing species with terrestrial adults (AcTe)**						
**Diptera: Tipulidae**						
Tipula sp.	0	0	2	2	0	0
**Diptera: Limoniidae**						
Eloeophila sp.	6	2	7	3	1	1
Molophilus sp.	1	1	1	1	0	0
Scleroprocta sp.	1	1	0	0	0	0
**Diptera: Pediciidae**						
Dicranota sp.	15	5	14	6	17	3
Tricyphona sp.	0	0	0	0	1	1
**Diptera: Empididae**						
Chelifera sp.	5	4	6	7	3	1
Clinocera sp.	3	1	0	0	0	0
Hemerodromia sp.	2	24	2	11	0	0
Wiedemannia sp.	10	5	3	4	2	1
**Diptera: Muscidae**						
Muscidae sp.	0	0	0	0	1	1
**Ephemeroptera**						
Ameletus inopinatus	11	10	12	7	18	7
Baetis muticus	3	97	12	54	20	30

Online supporting information

Metacommunity structuring in stream networks: roles of dispersal mode, distance type and regional environmental context
M. Grönroos, J. Heino, T. Siqueira, V.L. Landeiro, J. Kotanen & L.M. Bini

Baetis niger	15	78	10	12	10	6
Baetis rhodani	14	158	15	15	29	144
Baetis subalpinus	1	1	0	0	0	0
Centroptilum luteolum	0	0	1	1	0	0
Ephemera danica	0	0	4	4	0	0
Ephemerella aurivillii	9	11	8	19	15	4
Habrophlebia fusca	0	0	6	3	0	0
Habrophlebia lauta	0	0	12	6	0	0
Heptagenia dalecarlica	1	1	6	8	15	7
Heptagenia fuscogrisea	2	1	0	0	0	0
Heptagenia sulphurea	1	3	0	0	0	0
Leptophlebia marginata	9	7	6	2	0	0
Leptoplebia vespertina	4	21	0	0	0	0
Paraleptophlebia cincta	0	0	2	2	0	0
Paraleptophlebia submarginata	0	0	2	5	0	0
Parameletus chelifer	1	1	0	0	0	0
**Odonata**						
Somatochlora metallica	1	1	1	1	0	0
**Plecoptera**						
Amphinemura borealis	1	119	5	15	8	9
Amphinemura standfussi	0	0	1	2	1	1
Amphinemura sulcicollis	14	26	0	0	14	6
Arcynopteryx compacta	0	0	0	0	2	1
Capnia atra	1	2	0	0	3	1
Capnopsis schilleri	1	1	5	4	0	0
Diura bicaudata	3	1	3	1	2	2
Diura nanseni	7	2	4	2	0	0
Isoperla difformis	16	13	1	1	7	4
Isoperla grammatica	0	0	14	16	0	0
Isoperla obscura	0	0	5	2	0	0
Leuctra digitata	7	2	11	8	1	3
Leuctra fusca	1	1	0	0	0	0
Leuctra hippopus	2	2	0	0	19	5
Leuctra nigra	6	12	0	0	4	2
Nemoura sp.	16	29	18	49	28	37
Nemurella pictetii	3	26	0	0	1	1
Protonemura intricata	5	7	8	27	20	31
Protonemura meyeri	18	45	0	0	0	0
Siphonoperla burmeisteri	1	6	3	3	4	3
Xanthoperla apicalis	0	0	2	3	0	0
**Coleoptera**						
Agabus sp.	1	5	0	0	0	0
Bagous sp.	1	1	0	0	0	0
Colymbetes sp.	0	0	0	0	1	1
Dytiscidae sp.	1	1	0	0	0	0
Elmis aenea	18	42	14	22	10	7

Elodes sp.	3	1	4	3	0	0
Hydraena gracilis	13	13	8	17	2	1
Hydroporus sp.	0	0	0	0	1	1
Illybius sp.	2	10	0	0	0	0
Limnius volckmari	4	21	4	25	0	0
Oulimnius tuberculatus	5	6	7	8	1	1
**Megaloptera**						
Sialis fuliginosa	2	1	6	2	0	0
**Trichoptera**						
Agapetus ochripes	0	0	2	2	0	0
Apatania sp.	3	4	7	12	5	2
Arctopsyche ladogensis	0	0	0	0	1	1
Athripsodes sp.	0	0	1	14	0	0
Ceraclea annulicornis	0	0	2	11	0	0
Ceratopsyche silfvenii	1	1	1	4	0	0
Chaetopteryx sp.	0	0	5	2	0	0
Cheumatopsyche lepida	0	0	1	1	0	0
Halesus sp.	2	1	6	3	0	0
Hydropsyche angustipennis	0	0	7	1	0	0
Hydropsyche pellucidula	2	8	2	2	0	0
Hydropsyche saxonica	3	3	5	10	0	0
Hydroptila sp.	0	0	1	1	1	1
Lepidostoma hirtum	1	3	2	2	0	0
Limnephilus sp.	17	18	2	1	0	0
Micrasema gelidum	13	23	9	9	4	7
Micropterna sequax	0	0	1	1	0	0
Molannodes tinctus	0	0	3	2	0	0
Mystacides sp.	0	0	1	1	0	0
Neureclipis bimaculata	2	4	0	0	0	0
Philopotamus montanus	1	12	1	1	4	2
Plectrocnemia conspersa	10	14	8	9	3	1
Polycentropus flavomaculatus	0	0	7	8	0	0
Polycentropus irroratus	0	0	1	2	0	0
Potamophylax cingulatus	11	4	9	2	3	1
Rhyacophila fasciata	0	0	2	1	0	0
Rhyacophila nubila	6	5	10	5	23	5
Ryacophila obliterata	15	5	10	5	0	0
Sericostoma personatum	4	3	7	6	0	0
Silo pallipes	8	2	1	5	0	0

Online supporting information

Metacommunity structuring in stream networks: roles of dispersal mode, distance type and regional environmental context
M. Grönroos, J. Heino, T. Siqueira, V.L. Landeiro, J. Kotanen & L.M. Bini

Taking the information content of response datasets into account

The response matrices based on the dispersal mode groups (i.e., PaAq, PaTe and AcTe) differ in their information content ($IC = \sum P_i \times (1-P_i)$, where P_i is the proportion of sites occupied by the ith species; Table S3) due to the different number of species and patterns of rarity and commonness (see Lennon *et al.* 2004 and Siqueira *et al.* 2012). To take the likely effects of these differences in the information content into account, we randomly sampled species in each response matrix with high information content to obtain datasets (response matrices) with similar information contents (± 0.03).

Here, all PaAq datasets exhibited the lower information contents within regions (Table S3). Therefore, we randomly sampled species from PaTe and AcTe to create smaller datasets with similar information content (equal to PaAq ±0.03). We created 999 datasets representing the responses matrices for PaTe and 999 for AcTe and conducted the pRDA for each of these datasets. Then we calculated the mean and standard deviation (SD) for each resampled case (see Tables S4 and S5).

References

Lennon, J. J., Koleff, P., Greenwood, J.J.D., Gaston. K.J. (2004) Contribution of rarity and commonness to patterns of species richness. *Ecology Letters*, **7**, 81–87.

Siqueira, T., Bini, L.M., Roque, F.O., Marques Couceiro, S.R., Trivinho-Strixino S. & Cottenie, K. (2012) Common and rare species respond to similar niche processes in macroinvertebrate metacommunities. *Ecography*, **35**, 183–192.

Table S3: Number of species and information content (IC) within each dataset.

Dataset	Group	N species	IC
**All**	PaAq	23	1.72
	PaTe	112	8.43
	AcTe	93	9.12
**Iijoki**	PaAq	14	2.13
	PaTe	64	7.89
	AcTe	63	8.39
**Koutajoki**	PaAq	16	1.705
	PaTe	75	8.9
	AcTe	68	10.44
**Tenojoki**	PaAq	7	0.59
	PaTe	52	5.7
	AcTe	39	4.56

Online supporting information

Metacommunity structuring in stream networks: roles of dispersal mode, distance type and regional environmental context
M. Grönroos, J. Heino, T. Siqueira, V.L. Landeiro, J. Kotanen & L.M. Bini

Table S4. Adjusted R^2 values and p-values for each dispersal mode group and all taxa, in each basin and across basins based on observed data (obs.). Also the number of significant models (of the total of 999) when taking the information content into account is given in column ”*P,* n>0.05”. Fractions are as follows: [a+b] = environmental, [b+c] = spatial.

		[a+b]					[b+c]					
Basin	Group	Adj. R^2 obs.	*P* obs.		*P* n<0.05		Adj. R^2 obs.		*P* obs.		*P* n<0.05	
			Overl.	Waterc.	Overl.	Waterc.	Overl.	Waterc.	Overl.	Waterc.	Overl.	Waterc.
Iijoki	PaAq	0.028	0.280	0.260			0.060	0.064	0.160	0.058		
	PaTe	0.068	0.084	0.115	252	254	0.038	0.060	0.220	0.051	267	290
	AcTe	**0.174**	**0.005**	**0.005**	557	556	0.065	0.052	0.140	0.090	136	266
	All taxa	**0.15**	**0.005**	**0.005**			0.062	0.050	0.090	0.058		
Koutajoki	PaAq	0.08	0.170	0.090			0.014	0.039	0.410	0.200		
	PaTe	**0.109**	**0.017**	**0.005**	309	309	0.013	0.011	0.340	0.290	75	17
	AcTe	**0.107**	**0.015**	**0.013**	331	334	0.056	0.012	0.150	0.290	35	41
	All taxa	**0.12**	**0.005**	**0.005**			0.045	0.013	0.140	0.330		
Tenojoki	PaAq	-0.03	0.640	0.660			-0.103	-0.075	0.910	0.930		
	PaTe	0.028	0.190	0.150	139	136	**0.091**	**0.097**	**0.015**	**0.005**	296	388
	AcTe	**0.217**	**0.005**	**0.005**	423	427	-0.039	0.035	0.540	0.340	51	134
	All taxa	**0.115**	**0.005**	**0.020**			0.058	**0.095**	0.120	**0.020**		
All Basins	PaAq	**0.171**	**0.005**				0.067		0.005			
	PaTe	**0.201**	**0.005**		980		0.132		0.005		979	
	AcTe	**0.306**	**0.005**		999		0.136		0.005		998	
	All taxa	**0.278**	**0.005**				0.144		0.005			

Table S5. Pure fractions for each dispersal mode group and all taxa, in each basin and across the basins when using overland distances. Fractions and p-values are given based on observed data (obs.). The mean and standard deviation (SD) for each fraction and the number of significant models (n P<0.05) when taking the information content into account (999 resamplings) are given. Significant fractions are highlighted in bold. Fractions are as follows: [a] = pure environmental, [b] = shared, [c] = spatial, [d] = unexplained.

			[a]				[b]		[c]				[d]	
	Basin	Group	Adj. R^2 obs.	Adj. R^2 mean±SD	*P* obs.	*n* *P*<0.05	Adj. R^2 obs.	Adj. R^2 mean±SD	Adj. R^2 obs.	Adj. R^2 mean±SD	*P* obs.	*n* *P*<0.05	Adj. R^2 obs.	Adj. R^2 mean±SD
Overland	Iijoki	PaAq	0.050		0.26		-0.022		0.082		0.12		0.889	
		PaTe	-0.032	-0.001 ± 0.069	0.65	30	0.100	0.072 ± 0.05	-0.062	-0.001 ± 0.085	0.83	47	0.994	0.931 ± 0.106
		AcTe	**0.155**	0.144 ± 0.107	**0.03**	436	0.019	-0.004 ± 0.076	0.046	0.053 ± 0.066	0.20	97	0.780	0.806 ± 0.108
		All taxa	0.105		0.05		0.045		0.017		0.39		0.832	
	Koutajoki	PaAq	0.133		0.11		-0.053		0.067		0.21		0.853	
		PaTe	**0.134**	0.13 ± 0.098	**0.03**	367	-0.025	-0.045 ± 0.07	0.038	0.057 ± 0.072	0.33	90	0.853	0.857 ± 0.106
		AcTe	0.049	0.052 ± 0.099	0.20	143	0.058	0.04 ± 0.07	-0.002	-0.020 ± 0.091	0.5	25	0.896	0.928 ± 0.12
		All taxa	0.077		0.06		0.043		0.002		0.45		0.878	
	Tenojoki	PaAq	-0.046		0.71		0.016		-0.119		0.93		1.149	
		PaTe	**0.070**	0.065 ± 0.08	**0.03**	200	-0.042	-0.04 ± 0.064	**0.133**	0.147 ± 0.101	**0.01**	369	0.838	0.828 ± 0.115
		AcTe	**0.188**	0.138 ± 0.126	**0.03**	381	0.029	-0.03 ± 0.064	-0.068	0.043 ± 0.09	0.86	44	0.850	0.849 ± 0.106
		All taxa	**0.110**		**0.04**		0.005		0.053		0.17		0.832	
	All basins	PaAq	**0.102**		**0.01**		0.069		-0.002		0.51		0.831	
		PaTe	**0.074**	0.062 ± 0.027	**0.01**	860	0.127	0.094 ± 0.063	0.005	0.003 ± 0.009	0.105	121	0.794	0.84 ± 0.065
		AcTe	**0.164**	0.135 ± 0.052	**0.01**	979	0.142	0.118 ± 0.045	-0.006	-0.003 ± 0.006	1	31	0.700	0.75 ± 0.06
		All taxa	**0.131**		**0.01**		0.147		-0.003		0.84		0.725	

Table S6. Pure fractions for each dispersal mode group and all taxa, in each basin when using watercourse distances. Fractions and p-values are given based on observed data (obs.). The mean and standard deviation (SD) for each fraction and the number of significant models (n *P*<0.05) when taking the information content into account (999 resamplings) are given. Significant fractions are highlighted in bold. Fractions are as follows: [a] = pure environmental, [b] = shared, [c] = spatial, [d] = unexplained.

			[a]				[b]		[c]				[d]	
	Basin	Group	Adj. R^2 obs.	Adj. R^2 mean±SD	*P* obs.	*P* n<0.05	Adj. R^2 obs.	Adj. R^2 mean±SD	Adj. R^2 obs.	Adj. R^2 mean±SD	*P* obs.	*P* n<0.05	Adj. R^2 obs.	Adj. R^2 mean±SD
Watercourse	Iijoki	PaAq	-0.001		0.49		0.029		0.035		0.29		0.937	
		PaTe	0.032	0.057 ± 0.062	0.29	150	0.036	0.013 ± 0.05	0.024	0.044 ± 0.054	0.25	165	0.908	0.886 ± 0.073
		AcTe	**0.121**	0.103 ± 0.099	**0.04**	364	0.053	0.037 ± 0.05	-0.001	0.012 ± 0.047	0.54	56	0.827	0.847 ± 0.106
		All taxa	**0.112**		**0.02**		0.039		0.011		0.30		0.838	
	Koutajoki	PaAq	-0.004		0.52		0.084		-0.045		0.85		0.965	
		PaTe	**0.113**	0.091 ± 0.076	**0.02**	312	-0.004	-0.006 ± 0.03	0.015	-0.003 ± 0.037	0.27	17	0.876	0.918 ± 0.077
		AcTe	**0.121**	0.095 ± 0.078	**0.02**	289	-0.014	-0.004 ± 0.032	0.026	0.018 ± 0.037	0.15	21	0.867	0.89 ± 0.084
		All taxa	**0.120**		**0.01**		0.001		0.012		0.35		0.867	
	Tenojoki	PaAq	-0.104		0.95		0.074		-0.149		0.99		1.179	
		PaTe	**0.061**	0.049 ± 0.068	**0.03**	145	-0.032	-0.024 ± 0.061	**0.129**	0.12 ± 0.125	**0.01**	397	0.843	0.855 ± 0.123
		AcTe	**0.155**	0.124 ± 0.094	**0.03**	401	0.063	-0.015 ± 0.055	-0.028	0.062 ± 0.083	0.59	177	0.811	0.829 ± 0.098
		All taxa	**0.088**		**0.04**		0.027		0.068		0.06		0.817	
